# Evaluating enrichment use in group-housed rhesus macaques (*Macaca mulatta*): A machine learning approach

**DOI:** 10.1017/awf.2024.65

**Published:** 2024-12-09

**Authors:** Giulia Ciminelli, Claire Witham, Melissa Bateson

**Affiliations:** 1 Institute of Bioscience, Faculty of Medical Sciences, Henry Wellcome Building, Framlington Place, Newcastle University, Newcastle NE2 4HH, UK; 2 Centre for Macaques at Harwell, Medical Research Council, Salisbury, UK

**Keywords:** Animal welfare, computer vision, deep learning, enrichment, laboratory animals, macaques

## Abstract

Environmental enrichment programmes are widely used to improve welfare of captive and laboratory animals, especially non-human primates. Monitoring enrichment use over time is crucial, as animals may habituate and reduce their interaction with it. In this study we aimed to monitor the interaction with enrichment items in groups of rhesus macaques (*Macaca mulatta*), each consisting of an average of ten individuals, living in a breeding colony. To streamline the time-intensive task of assessing enrichment programmes we automated the evaluation process by using machine learning technologies. We built two computer vision-based pipelines to evaluate monkeys’ interactions with different enrichment items: a white drum containing raisins and a non-food-based puzzle. The first pipeline analyses the usage of enrichment items in nine groups, both when it contains food and when it is empty. The second pipeline counts the number of monkeys interacting with a puzzle across twelve groups. The data derived from the two pipelines reveal that the macaques consistently express interest in the food-based white drum enrichment, even several months after its introduction. The puzzle enrichment was monitored for one month, showing a gradual decline in interaction over time. These pipelines are valuable for assessing enrichment by minimising the time spent on animal observation and data analysis; this study demonstrates that automated methods can consistently monitor macaque engagement with enrichments, systematically tracking habituation responses and long-term effectiveness. Such advancements have significant implications for enhancing animal welfare, enabling the discontinuation of ineffective enrichments and the adaptation of enrichment plans to meet the animals’ needs.

## Introduction

Enrichment programmes are designed to provide stimulating and engaging experiences for animals in captivity, promoting their physical and psychological well-being. These programmes encompass a variety of modifications to the animals’ environment, collectively known as environmental enrichment. Facilities housing wild animals dedicate significant time and effort to plan, introduce, and evaluate enrichment programmes. However, not all enrichments are created equal, and careful consideration of the animals’ needs, biological nature, history, and the intended purpose of the enrichment is critical (Hare *et al.* 2007; Kemp 2023).

Despite the extensive literature on enrichment usage in captive settings for non-human primates (NHPs), the success of an enrichment strategy can be influenced by multiple factors, including enclosure structure, group dynamics, and the animals’ past experiences (Lutz & Novak 2005b). Therefore, enrichment strategy is not simply the introduction of novel toys or structures into the enclosure but requires careful planning and the establishment of a comprehensive programme that considers many aspects. A key part of enrichment programme design is deciding which type of enrichment to utilise. In this study we will focus on food-based and occupational enrichments.

Food-based enrichment tends to retain the animals’ interest and elicit an immediate response, keeping them engaged for long periods. Moreover, it promotes foraging behaviour and encourages natural activities (Sha *et al.* 2012). However, food-based enrichment must be carefully managed as providing additional food to the animals’ diet can lead to nutritional problems, such as obesity (Videan *et al.* 2007; Bauer *et al.* 2011). In addition, the continuous availability of food-based enrichments might make them less interesting or challenging for the animals over time, though presenting the food as enrichment can be more engaging than simply offering it as available food.

Occupational enrichments enable the primates to exhibit naturalistic behaviours, such as playing and visual and tactile exploration (Meehan & Mench 2007; de França Santos *et al.* 2022; Kemp 2023). However, animals may lose interest relatively quickly if a physical or occupational item becomes familiar and lacks novelty, unlike food-based enrichment (Paquette & Prescott 1988; Brent & Eichberg 1991; Line *et al.* 1991).

For these reasons it is necessary to assess the effectiveness of enrichment, regardless of its type. Monitoring the effectiveness of enrichment ensures that resources are being wisely invested and that the programme aligns with its intended goals (Newberry 1995). To ensure that the enrichment remains engaging to the animals it is important to evaluate the enrichment’s effectiveness both initially and over time for a comprehensive assessment.

Behavioural responses are commonly used to assess the effectiveness of enrichment, particularly since the introduction of stimuli often aims to promote specific behaviours (Hosey *et al.* 2013). However, collecting behavioural data from groups of captive animals can be complex and time-consuming, leading to inadequately tested enrichments (Swaisgood & Shepherdson 2005).

In recent years, advances in machine learning and computer vision have shown the potential of computers to solve some of these challenges. Artificial intelligence algorithms have replaced many human tasks, and several toolboxes based on computer vision have been implemented to identify body parts and objects in videos of humans and other animals (i.e. Bolya *et al.* 2019; Labuguen *et al.* 2019; Blanco Negrete *et al.* 2021). These approaches can gather more data, recording both the environment and the individuals within it and save time in data collection and analysis. In addition, computer vision-based technologies that rely on video recordings can remove the need for sensors or markers to detect and track the animals, making them non-invasive and non-intrusive.

Despite the rising availability of machine learning algorithms and increasing interest from the animal behaviour research community, there are still many challenges associated with using computer vision methodologies to extract valuable data. One major limitation is that NHPs, like many other species, may assume unusual postures, have similar physical features, or be obscured by objects or structures in their environment (Vidal *et al.* 2021).

Our aim is to validate and utilise automated methodologies based on deep learning to assess enrichment use in a breeding colony housing rhesus macaques (*Macaca mulatta*). Rhesus macaques participate extensively in research due to their genetic and physiological similarities to humans, making them valuable for translational studies (Kalin & Shelton 1989). Recordings of group-housed macaques will be used to develop the automated pipelines capable of collecting data on enrichment usage.

Specifically, we will evaluate the effectiveness of two different types of enrichment:

- A food-based enrichment where the container is continually present in the enclosure but only filled with food once a week, aiming to assess its effectiveness and how the usage changes depending on whether the container is empty or full;
- A cognitive and occupational enrichment, specifically introduced for this study, with the objective of detecting changes in macaques’ interest in it over time.

## Materials and methods

### Facility and subjects

This study is based on enrichment provided at the Centre for Macaques (CFM), UK, where rhesus macaques were housed in socially structured breeding groups consisting of one adult male, multiple females, and their offspring. Most juvenile animals were weaned (permanently separated from their mothers) between 12 and 36 months of age and moved to same-sex stock groups with monkeys of a similar age (a small number of female juveniles remained with their natal group for breeding). The age range across the groups was 0–16 years and the weight ranged from 0.4 kg for newborn infants to 19.5 kg for the largest male macaque. Each group was composed of up to 18 individuals and were housed in enclosures featuring a playpen area with wood-shavings on the floor (8.04 m × 3.35 m × 2.8 m; length × width × height) and an adjoining caging area (6.12 m × 1.5 m × 2.8 m). Multiple hatches at different heights joined the two areas together. The playpen area was equipped with a range of structure-based enrichment items including platforms at different heights, vertical and horizontal poles and fire hoses. The pens were cleaned once a fortnight.

Once a day the animals were fed with a complete primate diet (currently supplied by International Product Supplies, UK, formerly supplied by Special Diet Services), two types of vegetables and fruit (on one day each week one of the vegetables is replaced by hard-boiled eggs) and a scattered forage mix of grains and seeds. Feeding times were typically between 0800 and 1000h each day.

### Enrichment items

**Figure 1.**
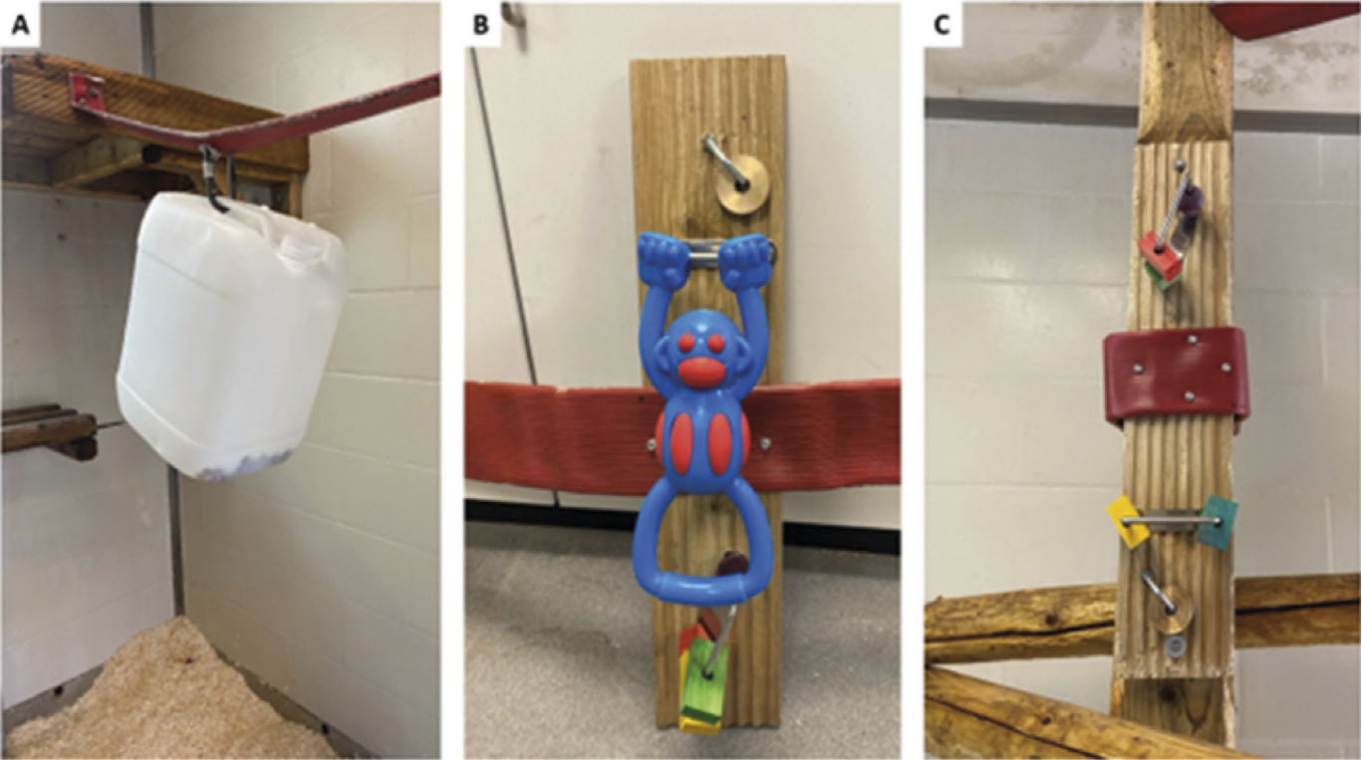
Types of enrichment used for the studies showing (A) white drum used for food enrichment, (B) puzzle enrichment with blue monkey toy and (C) revised puzzle enrichment without blue monkey toy.

For this study we focused on two specific enrichments: a white drum filled with raisins which was used as a food-based enrichment (Figure 1A) and an occupational puzzle (Figure 1B, 1C).

The white drum was suspended with a carabiner from one of the horizontal poles in the playpen and was filled with raisins every Monday morning during the regular feeding time for the macaques (Figure 1A). The drum featured a larger hole in the top and small holes in the bottom, prompting the macaques to employ various methods to extract the raisins. They may either shake and overturn the drum from above or access it from below while positioned on the ground. Raisins were a favoured treat, reserved mostly for training and human habituation exercises. The quantity of raisins distributed within the drum corresponds to the size of the macaque group. Typically, the macaques empty the drum within a few hours. The container was always present in the playpen and was already an established part of the enrichment plan when this study commenced. Although it was not possible to determine the exact date of its introduction due to different timelines, schedule changes, and group variations, the white drum was in each group for more than two months prior to the start of the study, ensuring it was no longer a novelty for the animals.

The cognitive puzzle was attached to one of the vertical poles in the playpen (Figure 1B, 1C). Initially, it consisted of three metallic U-shaped bolts, one of which had a metal circle attached, the second has a blue dog toy depicting a monkey (blue monkey), and the third had up to four pieces of coloured wood of various shapes (wooden blocks designed for pet birds; Figure 1B). However, during the study (the fourth group to be tested), the blue monkey became damaged in the first few days of the trial and was subsequently replaced with several more pieces of coloured wood of different shapes (Figure 1C). The puzzle was intentionally designed to encourage macaques to manipulate the metal circle and wooden block around the metal hooks, providing them with exposure to varied materials, colours, and textures. In the case of the blue monkey, it afforded greater movement options because it was attached solely from its top side, and its material properties permitted the macaques to pull and twist it. This enrichment was introduced into the enclosure specifically for the purpose of the study and had not been previously presented to the macaques.

### Data collection

**Figure 2.**
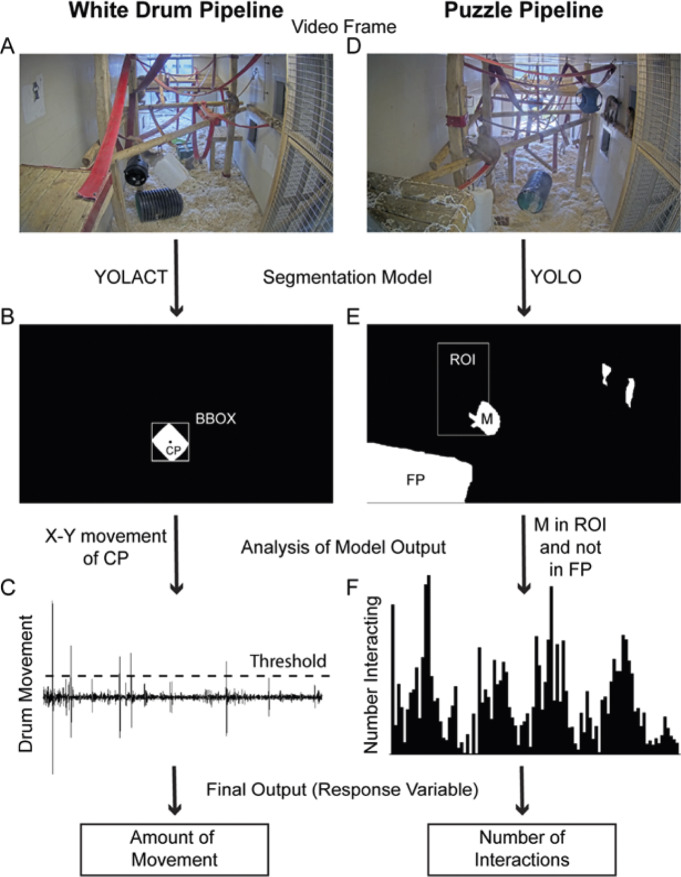
Automated pipelines used to detect enrichment usage by groups of macaques. These figures illustrate the steps involved in the automated detection of white drum enrichment usage (A to C) and puzzle enrichment usage (D to F). More specifically these show (A) the input frame from video for white drum enrichment, (B) segmentation mask and bounding box (bbox) for the white drum (circle shows the centre-point [CP] of the bounding box, (C) example of x co-ordinate from the bounding box with a threshold (dotted line), (D) input frame from video for puzzle enrichment, (E) segmentation masks for monkeys (M and unlabelled white objects) and front platform (FP) together with region of interest (ROI) and (F) number of monkeys interacting with the puzzle enrichment.

Video for both studies was recorded from CCTV cameras mounted at the top of the main window into each play pen (Axis P1435-LE cameras [Axis Communications, Sweden] recording continuously to an Axis Camera station). These cameras gave a good video of the playpen (for an example, see Figure 2). For the first study (white drum) the model was trained on a Dell XPS machine (Dell Technologies, UK) with a Nvidia Geforce GTX 1070 graphics card (Nvidia Corporation, USA) and the recorded videos were analysed on a standard commercial laptop (XPS-15 with a Nvidia Geforce GTX 1650 Ti card); both running Windows® 10. For the second study (cognitive puzzle) the model was trained, and the videos analysed on a Scan Systems 3XS Deep Learning DBP G2-18C machine (Scan Computers International, UK) with two Nvidia Geforce RTX 3080 Turbo v2 cards, running Ubuntu 22.04.

#### Data collection for white drum enrichment study

This study involved a dataset comprising information from nine different macaque groups, consisting of five breeding groups (BG) and four juvenile groups (JG), with a mean (± SD) group size of 10 (± 2.9) animals. Each group was observed and recorded for a total of three Mondays (when the white drum contained raisins) and three Thursdays (when the drum was empty) for three weeks (these were non-cleaning weeks to avoid cage cleaning affecting the results). Mondays and Thursdays were chosen as the staff working hours on those days were consistent (staffing is reduced on the weekends) but also to give time for the monkeys to completely empty the container (it was always empty by Thursday). The observation period each day started from the time the monkeys were fed until 6 h later.

#### Data collection for puzzle enrichment study

This study involved a dataset comprising information from twelve different macaque groups, consisting of six breeding groups (BG) and six juvenile groups (JG), with a mean (± SD) group size of 10 (± 2.8).

The animals were confined to the cage room for ~15 min whilst the enrichment was installed on one of the vertical poles in the main playpen (see Figure 1C). Each group was observed and recorded for a total of 27 days after the puzzle enrichment was introduced to the playpen. Each observation period lasted for 12 h per day, starting at 0700h (the time the artificial lighting comes on). Days involving pen cleaning were omitted from the datasets. During cleaning the animals are temporarily confined to the cage room, reducing the available time for interacting with the enrichment on those days.

### Automated methodologies

Computer vision provides a range of deep-learning-based methods for object detection (locating where the object is) and object recognition (identifying what the object is). For this study we used two different deep-learning frameworks both of which are derived from the YOLO (You Only Look Once) framework (Jocher *et al.* 2023). In both cases these are object segmentation-based methods. Traditional object detection methods draw a box around the object of interest (e.g. the box shown in Figure 2B). Object segmentation models draw an outline around the object of interest (see the white masks in Figure 2E). Object segmentation has several advantages over traditional object detection, particularly when detecting animals which can adopt many different postures such as monkeys.

For both methods the same image labelling, training and deployment process was used to create robust models:

1. A random selection of stills from the video cameras to produce a ground-truth dataset for training the model;
2. Humans label the objects in the images using image labelling software;
3. Train the object detection model (YOLACT or YOLO);
4. Check the performance of the model. At this stage it may be necessary to label more images and retrain the model;
5. Deploy the model on the videos (example frames in Figure 2A, 2D) and get an output of object location (bounding box, segmentation mask and object class; examples shown in Figure 2B, 2E); and
6. Analyse the output to get a meaningful measure (e.g. movement of object or overlap between the monkeys and a region of interest; examples shown in Figure 2C, 2F).

#### Automated methodology for white drum enrichment

For the first study we used YOLACT (You Only Look At CoefficienTs) (Bolya *et al.* 2019) to locate and track the movement of the white drum. The model was trained to segment the drum from the background (Figure 2A). Since the white drum is distinctive in colour and fixed in shape, its detection was much simpler than the detection of the monkeys and therefore less time needed to be spent labelling images and training the model. A total of 292 images were labelled (randomly extracted from a subset of the whole dataset of videos recording the macaques interacting with the drum). These were randomly split with 70% used for the training dataset and 30% for the validation dataset. We then trained a YOLACT model (with the default settings). To test the ability of the model to generalise, we excluded one group from the training set. The model precision was acceptable with an intersection over union of 82% (IoU; see Table 1).

**Table 1.** The metrics used to validate the White Drum Model, which was employed to detect the enrichment across nine rhesus macaque enclosures. Specifically, it reports the mean average precision (mAP) which compares the ground-truth bounding box (and mask) to the detected box (and mask) and returns a score. These comparisons are reported for different intersection over union (IoU) thresholds. This is a measure of the overlap between the predicted bounding box (and mask) and the ground truth bounding box (and mask)

	All	0.50	0.60	0.70	0.80	0.90
Box	76.7	100	100	98	88.8	20.1
Mask	82.5	100	100	94.2	92.1	59.5

From each detection we extracted the centre x,y co-ordinates of the drum (Figure 2B; label ‘CP’) and conducted a frame-by-frame analysis to look for differences in these centre-point co-ordinates to determine whether the drum was moving or not (Figure 2C).

To test the assumptions that using item movement was a good proxy for its usage we compared the analysed model output with the ground truth. The ground truth data were obtained by manually analysing videos using BORIS (Friard & Gamba 2016) which allowed for the collection of the actual time the macaques spent interacting with the item. For this validation, six videos were randomly selected from various macaque groups, and a manual analysis was performed on 111,465 frames. These manual analyses recorded the time the animals spent interacting with the drum, both when it was moving and when it was not, collecting all the intervals of interaction. The model output had an accuracy of 99% in assessing the enrichment usage (see Table 2).

**Table 2.** The evaluation of the white drum enrichment pipeline, reporting the accuracy, recall, and precision in detecting the white drum usage. These metrics compare the pipeline output with the ground truth, obtained from direct observation of six videos recording macaques interacting with the enrichment

Number of videos	6
Number of frames	111,465
True positive (TP)	1,150
True negative (TN)	110,309
False positive (FP)	146
False negative (FN)	198
Accuracy in assessing enrichment usage 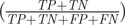	99%
Recall in assessing enrichment usage 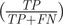	85%
Precision in assessing enrichment usage 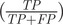	88%

#### Automated methodology for puzzle enrichment

For the second study a new improved version of YOLO (YOLO v8; Ultralytics) was released shortly before the study began. As this had several improvements over the YOLACT framework, including ease of use, we switched from YOLACT to YOLO v8 for the second study. The YOLO model was trained to segment 26 different objects from the background including the monkeys themselves, the various platforms and the puzzle enrichment. A different approach was needed for measuring interaction with the puzzle enrichment as the movement involved with the puzzle objects was mostly small (and often obscured by the monkey’s hand or body). Therefore, we focused on detecting interactions by concentrating on the monkeys, the front platform, and the puzzle enrichment. Monkeys were considered to be interacting with the puzzle enrichment if their segmentation mask (labelled ‘M’ in Figure 2E) overlapped with the region of interest for the puzzle enrichment (box labelled ‘ROI’ in Figure 2E) but did not overlap with the front platform (mask labelled ‘FP’ in Figure 2E). The two unlabelled masks in Figure 2E are monkeys that were not interacting with the enrichment.

A total of 258 labelled frames (randomly selected from the CCTV footage) were used to train the YOLO v8 model split between 70% training images, 15% validation images and 15% test images. The YOLOv8 model underwent training to identify macaques, the wooden platform situated at the front of the enclosure, the blue monkey and the wooden blocks on the enrichment puzzle (Figure 1B, 1C, Figure 2D, 2F). The precision-recall curves and mean average precision give a measure of how accurate the model was at detecting and locating the objects (Table 3). Notably, as demonstrated in the table below, objects closer to the cameras were detected with greater accuracy. This distinction proved valuable, as our focus was primarily on macaques interacting with the puzzle in the foreground. Conversely, those situated towards the rear of the enclosure, near the window, were not within the scope of this study, and were more prone to being mistaken for background elements (see Figure 2) due to their distance from the camera presenting a challenge even for a human observer.

**Table 3.** The validation results of the YOLO model used to evaluate puzzle enrichment usage across twelve macaque groups, including precision, recall, and Mean Average Precision (mAP) for various objects

	Precision	Recall	Mean average precision
Macaque	0.798	0.466	0.338
Front platform	0.904	0.875	0.851
Blue monkey	0.861	0.725	0.625
Wooden block	0.787	0.456	0.297

Additionally, stable objects such as the blue monkey and the front platform exhibited the highest levels of accuracy (83 and 92%, respectively; see Table 3).

Due to unsatisfactory wooden block detection (57% accuracy; see Table 3), any macaque engaging with the enrichment was considered within the region of interest (ROI) surrounding the enrichment. Moreover, as the enrichment was positioned behind the front platform, to prevent counting macaques on the platform as individuals within the ROI, those whose area overlapped with the front platform were excluded from the count of individuals interacting with the enrichment.

The object detection model captures the count of interacting macaques with the enrichment in a single frame per second, recorded at a frame rate of 15 frames per second. These results are a substantial dataset, yielding a total of 3,600 data-points per hour of video. To streamline this dataset, an R script was employed to calculate the number of macaques engaging with the enrichment for each hour of observation.

The pipeline’s accuracy was further assessed by comparing the number of macaques detected within the ROI with those identified by a human observer. In this analysis, one video was randomly selected from each of the 12 groups. For a total of 13 frames for each video, taken at hourly intervals, both the model and the human observer independently counted the number of individuals within the ROI. The model achieved an accuracy of 87.5% in detecting macaques within the ROI (for more details see Table 4).

**Table 4.** The validation of the puzzle enrichment pipeline used to evaluate the puzzle enrichment usage in twelve different groups of rhesus macaques. This reports the accuracy of the model in detecting the macaques in the region of interest (ROI) around the enrichment puzzle

Number of videos	12
Number of frames	156
True positive (TP)	45
True negative (TN)	102
False positive (FP)	10
False negative (FN)	11
Accuracy in detecting the macaques in the ROI 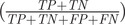	87.5%

### Statistical analysis

To analyse the output data from the two pipelines, we utilised R Studio. For all statistical analyses an alpha level of 0.05 was set.

#### White drum enrichment

In this study, a linear mixed model analysis was employed to examine the data, allowing for the incorporation of both fixed and random effects to account for potential sources of variation within the dataset. The lmer function from the lme4 package was used to model the interaction data (Bates *et al.* 2014).

The outcome variable was the number of frames where the drum was observed to be moving. To address potential missed detections (false negatives), the count of frames where the object was detected by the algorithm was included as a covariate in the model. The independent variable of interest was the day of the week, specifically comparing Mondays versus Thursdays.

The model incorporated the following fixed factors:

- The hours following feeding time were categorised into intervals: 1 h, 2 h, 3 h, 4 h, 5 h, and 6 h post-feeding;
- Group type, distinguishing between breeding groups and juvenile groups.

Additionally, group identity was treated as a random factor in the model.

#### Puzzle enrichment

A generalised linear mixed model analysis, following a Poisson distribution, was employed to examine the data, allowing for the incorporation of both fixed and random effects to account for potential sources of variation within the dataset. The glmer function from the lme4 package was used to model the interaction data (Bates *et al.* 2014). In this study, the dependent variable was the number of detected monkeys interacting with the item each hour and the independent variables were the days since the enrichment was added to the group, the time of day and the presence/absence of the blue monkey. The model included group type (breeding group or juvenile group) as a fixed effect, group size as an offset and group identity as a random factor.

### Ethical approval

The data collection took place at the Medical Research Council’s ‘Centre for Macaques’, which adheres to the regulatory standards set by the UK Home Office for housing captive non-human primates. For the introduction of the enrichment items, approval was obtained during the Centre for Macaques AWERB meeting held on March 13, 2023 (approval number: CFM2023E001). Since all the studies were observational in nature, no additional licensing was necessary. The study is part of a PhD project that was ethically approved by the Newcastle University Animal Welfare and Ethical Review Body.

## Results

### White drum study

The object detection-based pipeline yielded significant findings in number of frames where the drum was observed to be moving between Mondays, when the white drum was filled with raisins, and Thursdays, when the white drum remained empty. In total, the study accumulated 324 h of data, which were subsequently analysed using an automated pipeline over a span of three days.

**Figure 3.**
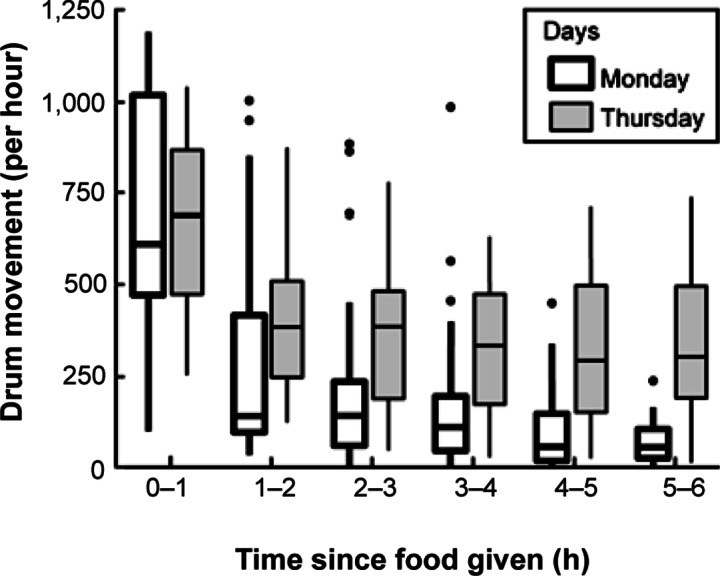
White drum enrichment use across nine macaques groups, each with a mean (± SD) group size of 10 (± 2.9) individuals. Boxplots showing the number of frames where the drum moved, caused by macaques’ integration with it, on Mondays and Thursdays for each interval after food was given. Notably, raisins were only placed into the drum on Mondays, while feeding time occurred in the morning every day.

The amount of drum movement was significantly higher (*F*_5,308_ = 41.4; *P* < 0.001) during the first hour after feeding time compared to the subsequent hours (Figure 3, Table 5). However, no significant difference (*F*_1,7_ = 0.4; *P* = 0.5; Table 5) in item interaction was observed between breeding groups and juvenile groups. The number of detections was included in the model to control for the impact of missed detections. For each additional detection of the object the movement is expected to increase by 0.73 units (*F*_1313_ = 6.7; *P* = 0.01; Table 5), showing the importance of controlling for missed detections.

**Table 5.** Type III Analysis of Variance (ANOVA) Table with Satterthwaite’s Method, showing the effects of detection per hour, hour of the day, group type, and weekday on the white drum enrichment movement across nine groups of macaques over a total of 324 h

Effect	Sum Sq.	Mean Sq.	Num DF	Den DF	F-value	*P*(>F)
Detection per hour	292,378	292,378	1	313	6.7	0.009
Hour	8,996,050	1,799,210	5	308	41.4	< 0.001
Group type	18,857	18,857	1	7	0.4	0.53
Weekday	1,739,509	1,739,509	1	308	40.1	0.008

The results show that the interaction with the container was highest during the first hour after feeding and then gradually decreased in the subsequent hours. Surprisingly, the overall item movement was significant higher (*F*_1,308_ = 40.1; *P* = 0.008; Table 5) on Thursdays when the drum was empty (Figure 3; shaded bars).

### Puzzle enrichment

**Figure 4.**
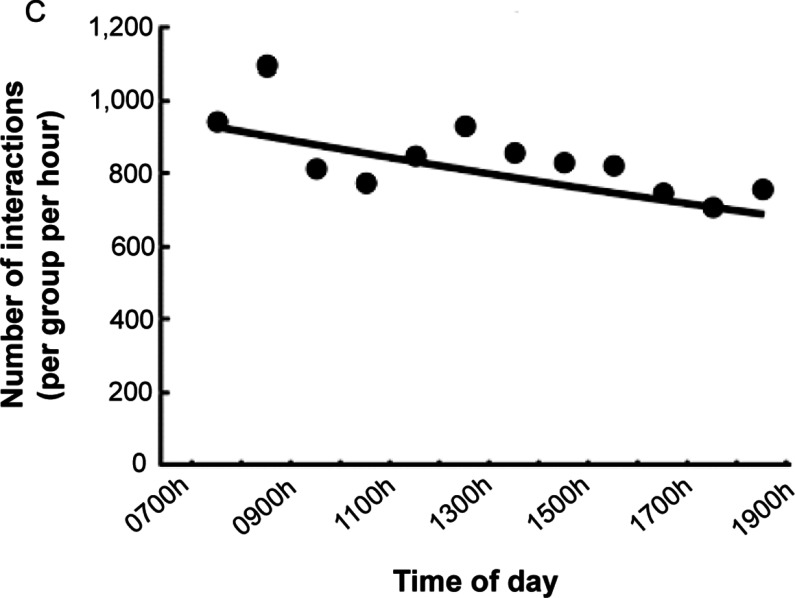
Interaction with puzzle enrichment by twelve groups of macaques each with a mean (± SD) group size of 10 (± 2.8) individuals showing (A) average number of interactions with the puzzle enrichment per group per hour plotted against days since added (solid line shows fit from the mixed effect model output), (B) average number of interactions with the enrichment per group per hour when the blue monkey toy was present or absent and (C) average number of interactions with the enrichment per group per hour relative to time of day (solid line shows fit from the mixed effect model).

The object detection-based model analysed 300 days of observations for a total of 3,612 h of data and required just a single hour to analyse the content of a whole day of video recordings. There was a declining pattern in the interaction with the puzzle across the 28-day period where the enrichment was present (β = –0.021, SE = 0.001, z = –297.52; *P* < 0.001; Figure 4A, Table 6). There was also a significant increase in puzzle engagement for the variant containing the blue monkey, compared to the one featuring only wooden blocks (β = 0.68, SE = 2.2, z = 3.08; *P* = 0.002; Figure 4B, Table 6). The hour of the day also influenced the enrichment usage, with the puzzle being used less during late evening hours (β = –0.027, SE = 0.0002, z = –163.24; *P* < 0.001; Figure 4C, Table 6).

**Table 6.** Coefficients and Statistical Significance for the puzzle enrichment study, which aimed to detect enrichment usage across 12 groups of rhesus macaques over a month of observation. The table presents the estimates, standard errors, z-values, and *P*-values for each predictor variable in the model, indicating the effect size and significance of each variable

	Estimate	Std Error	Z value	*P*(>z)
(Intercept)	4.71	0.14	34.16	< 0.001
Days since added	–0.021	0.001	–297.52	< 0.001
Group type-juvenile	0.12	0.19	0.62	0.53
Time of day	–0.027	0.0002	–163.24	< 0.001
Blue monkey - Yes	0.68	2.22	3.08	0.002

## Discussion

In this study our goal was to monitor how groups of rhesus macaques interacted with enrichment items in a breeding colony. We sought to streamline this process by employing machine learning technologies. Specifically, we focused on two types of enrichment: a food-based enrichment and an occupational puzzle. We developed two separate pipelines to assess the usage of these enrichments across a total of 21 groups of rhesus macaques in the breeding colony.

Both automated models demonstrated the capability to automatically detect macaque interactions with enrichments. The approaches, one focusing on detecting the white drum and tracking its movements, and the other identifying macaques in close proximity to the puzzle enrichment, exhibited high accuracy.

### Food-based enrichment (white drum)

It is well-documented that repeated exposure to a constant object in an animal’s environment can result in decreased interest compared to an intermittent object (Kemp 2023). The phenomenon behind this reduced interest is habituation (Gallistel 1990; Kuczaj *et al.* 2002). We did not measure drum movement at the time of its initial introduction. Therefore, we cannot determine whether habituation occurred. However, our results show that even with the extended presence of the drum in the enclosures, the macaques continue to actively engage with and utilise the enrichment. It is important to highlight that the drum is replenished subsequent to feeding sessions. On both days, irrespective of its contents, there was an elevated level of engagement shortly post-feeding compared to subsequent hours. However, in instances where the item was empty, macaque interaction surpassed that observed when it was filled. This heightened interaction during emptiness may be attributed to intensified efforts in extracting food from the drum, potentially stemming from the frustration induced by its empty state even after the designated feeding period. In fact, even though the drum is not removed when empty, this behaviour could be associated with a form of enrichment removal, which is known to be particularly frustrating and can reduce the satisfaction derived from low-reward enrichment (Amsel 1958; Papini 2003; Latham & Mason 2010). Since the animals at CFM consistently live in enriched enclosures, this change in how the white drum enrichment is presented is unlikely to impact their overall welfare (Latham & Mason 2010).

While one may posit that if the drum consistently contains raisins only on Mondays, the animals at CFM should anticipate this routine, empirical evidence suggests that regular feeding times do not necessarily render routines reliably predictable (Waitt & Buchanan-Smith 2001). The anticipation of food appears to be elicited by cues preceding the feeding event, indicating that animals may form associations beyond strict temporal patterns (Waitt & Buchanan-Smith 2001). In essence, if the animals come to link the presence of raisins in the white drum with feeding time, an expectation of its replenishment on a daily basis may arise.

In addition, when the drum is full, some monkeys may tend to monopolise it, limiting access for others; however, when it’s empty, a broader range of monkeys may engage with it as individuals leave upon discovering it’s empty. This increased turnover of monkeys engaging with the enrichment could also explain the heightened activity observed on Thursdays. In summation, the white drum enrichment continues to captivate the macaques at CFM, eliciting species-specific behaviours, including manipulation and foraging. Importantly, these behaviours persist even in the absence of high-caloric food items like raisins, suggesting that the enrichment itself may serve as a stimulating factor independent of daily food provision.

## Occupational enrichment (puzzle)

In contrast to food-based enrichment it is well-known that straightforward toys experience a rapid decline in usage among NHPs (Bayne *et al.* 1993; Lutz & Novak 2005a; Kemp 2023). Therefore, when considering non-food-based enrichment, the ability to monitor animals’ interest in these items is paramount. The employment of this automated pipeline enabled the detection of an initial decline in interest towards the puzzle in the majority of groups. Additionally, factors such as destructibility, complexity, physical alterability, texture, manipulability, colour, and size of the objects may influence their utility (Pruetz & Isbell 2000). Our model successfully discerned disparities among various types of puzzles, illustrating how the presence of a brightly coloured rubber toy (i.e. the blue monkey) amplified interactions with the puzzle. The blue monkey was brightly coloured and stood out distinctly from the neutral-toned wooden blocks, both in texture and appearance. Its design, featuring distinctive facial features like eyes, added an extra layer of visual interest. This observation suggests that puzzles with more complexity and a variety of toys might be more engaging for the macaques (Schapiro & Bloomsmith 1995; Kemp 2023).

The pipeline shows a decrease in number of interactions with the puzzle during feeding time (0800–0900h) and in the afternoon/evening (1500–1800h) while the macaque group is known to be resting. In addition, the pipeline brought to light a notable decline in puzzle usage, particularly during late afternoons. This may be due reduced staffing levels and a generally quieter atmosphere at CFM during these times or because rest periods in the afternoon are common. Existing studies on non-human primates in zoo environments consistently demonstrate that their behaviour is influenced by human presence (Hosey & Druck 1987; Chamove *et al.* 1988; Wells 2005). Specifically, these animals tend to exhibit increased activity and spend more time near the front of their enclosures when there is a greater influx of visitors. Conversely, in times of low human presence, they tend to allocate more time to rest and relaxation (Wells 2005). This dual effect likely contributes to the late afternoon reductions in enrichment interaction observed at CFM, coinciding with lower staff presence. Future studies using the same YOLOv8-based pipeline would be able to gather more precise data on the positions of macaques within the playpen, facilitating an examination of whether the animals are indeed allocating more time to established resting zones, such as the front and back platforms.

Despite studies suggesting that young animals tend to exhibit greater interest in novelty and enrichment (Schapiro & Bloomsmith 1995; Schapiro *et al.* 1996; Lutz & Novak 2005a), the current project did not yield any significant difference in enrichment interaction between group types. This could potentially be attributed to the presence of young individuals within the breeding group as well as in the juvenile groups.

### Animal welfare implications

Using these pipelines, we evaluated an existing enrichment to determine its continued use and assessed the impact of a new enrichment, including identifying when it no longer interested the animals. This advancement is significant for macaque welfare, as enrichment planning and evaluation are crucial to ensure that the provided enrichments fulfil their intended purpose. These technologies enabled us to effectively assess enrichment usage, ensuring that the planning and implementation of enrichment programmes are thoroughly evaluated, with the animals’ needs always prioritised.

## Conclusion

In conclusion, the utilisation of these automated pipelines allows for a significant advancement in data collection and analysis. Not only do they enable the detection of macaque interactions with enrichments, but they also facilitate comparative analyses of various enrichments and their impact on different group types. Furthermore, the results can be harnessed to enhance the management of the enrichment programme and formulate a more effective strategy tailored to the specific requirements and preferences of each group.

## Supporting information

Ciminelli et al. supplementary material Ciminelli et al. supplementary material

## Supplementary material

The supplementary material for this article can be found at http://doi.org/10.1017/awf.2024.65.

## Research data for this article

The datasets for the output of the automated datasets are available in the Supplementary material (in CSV file format). The R and Python scripts are available at https://github.com/GiuliaCiminelli/AutomatedMacaqueBehaviour, together with the Puzzle Enrichment model. The white drum model is available upon request.
