# Supplementary material for: Evaluating enrichment use in group-housed rhesus macaques (Macaca mulatta): A machine learning approach
Source: Anim Welf. 2024 Dec 9;33:e59. doi: 10.1017/awf.2024.65 (PMC11655280; doi:10.1017/awf.2024.65)
Supplement: Ciminelli et al. supplementary material [file S0962728624000654sup001.pdf]

Supplementary material

Evaluating enrichment use in group-housed rhesus macaques (*Macaca mulatta*): A machine learning approach

Giulia Ciminelli https://orcid.org/0000-0002-3773-0643[1], Claire Witham https://orcid.org/0000-0003-1182-0074[2], Melissa Bateson[1]

[1] Institute of Bioscience, Faculty of Medical Sciences, Henry Wellcome Building, Framlington Place, Newcastle University, Newcastle NE2 4HH, UK

[2] Centre for Macaques at Harwell, Medical Research Council, Salisbury, UK

Author for correspondence: Giulia Ciminelli, email: giuliaciminelli88@gmail.com

The R and Python scripts used for detecting enrichment items, extracting interaction data, and performing statistical analyses, along with the necessary Excel files containing relevant information, are available at AutomatedMacaqueBehaviour .

In the GitHub repository there are two folders of interest:

- Enrichment_Yolcat: This folder contains Python and R scripts designed to extract movement data using a YOLACT-based model trained to recognize an enrichment item. Additionally, it includes an Excel file with feeding time data, which is required for the R script and subsequent statistical analyses.

- Enrichment_Yolo: This folder contains Python and R scripts for extracting data on the number of macaques interacting with an enrichment puzzle. The YOLO-based model is located in the 'Model' sub-folder. Additionally, there is an Excel file with group information, including size and type, which is required for the statistical analyses performed by the R script.
